# Enzymatic Degradation of Cortical Perineuronal Nets Reverses GABAergic Interneuron Maturation

**DOI:** 10.1007/s12035-022-02772-z

**Published:** 2022-03-01

**Authors:** Ashleigh Willis, Judith A. Pratt, Brian J. Morris

**Affiliations:** 1 Institute of Neuroscience and Psychology, College of Medical Veterinary and Life Sciences, University of Glasgow, Glasgow, G12 8QQ UK; 2 Strathclyde Institute of Pharmacy and Biomedical Sciences, University of Strathclyde, Glasgow, G4 0RE UK

**Keywords:** c‐Jun N‐terminal kinase (JNK), Extracellular matrix, TAOK2, Brain‐derived neurotrophic factor (BDNF), Bicuculline, Synaptic plasticity, Glycoprotein biosynthesis

## Abstract

Perineuronal nets (PNNs) are specialised extracellular matrix structures which preferentially enwrap fast-spiking (FS) parvalbumin interneurons and have diverse roles in the cortex. PNN maturation coincides with closure of the critical period of cortical plasticity. We have previously demonstrated that BDNF accelerates interneuron development in a c-Jun-NH_2_-terminal kinase (JNK)–dependent manner, which may involve upstream thousand-and-one amino acid kinase 2 (TAOK2). Chondroitinase-ABC (ChABC) enzymatic digestion of PNNs reportedly reactivates ‘juvenile-like’ plasticity in the adult CNS. However, the mechanisms involved are unclear. We show that ChABC produces an immature molecular phenotype in cultured cortical neurons, corresponding to the phenotype prior to critical period closure. ChABC produced different patterns of PNN-related, GABAergic and immediate early (IE) gene expression than well-characterised modulators of mature plasticity and network activity (GABA_A_-R antagonist, bicuculline, and sodium-channel blocker, tetrodotoxin (TTX)). ChABC downregulated JNK activity, while this was upregulated by bicuculline. Bicuculline, but not ChABC, upregulated *Bdnf* expression and ERK activity. Furthermore, we found that BDNF upregulation of *semaphorin-3A* and IE genes was TAOK mediated. Our data suggest that ChABC heightens structural flexibility and network disinhibition, potentially contributing to ‘juvenile-like’ plasticity. The molecular phenotype appears to be distinct from heightened mature synaptic plasticity and could relate to JNK signalling. Finally, we highlight that BDNF regulation of plasticity and PNNs involves TAOK signalling.

**Supplementary Information:**

The online version contains supplementary material available at 10.1007/s12035-022-02772-z.

## Introduction

Perineuronal nets (PNNs) are complex extracellular matrix structures which preferentially surround the soma, proximal dendrites and axonal nodes of Ranvier of specific neuronal subtypes [1–5]. These nets are predominantly found around fast-spiking parvalbumin (PVB) interneurons [6], although a small number of exceptions have been reported (notably, a subset of somatostatin (SST) interneurons which express Kv3 potassium channels [7] and a population of glutamate-reactive neurons which are surrounded by diffuse PNNs [8]).

PNNs possess a sophisticated molecular composition, with some work indicating heterogeneity of these structures across brain regions and cell types [9, 10]. Nonetheless, it appears that PNNs share a basic molecular composition [5]. Structurally, PNNs are composed of a hyaluronic acid (HA) backbone which is enriched with chondroitin sulphate proteoglycans (CSPGs; predominantly aggrecan (*Acan*/ACAN), brevican (*Bcan*/BCAN), neurocan (*Ncan*/NCAN) and versican (*Vcan*/VCAN)) bound via glycosaminoglycan sidechains [5, 11–13]. PNNs are stabilised by tenascin-R (TN-R) and hyaluronan and proteoglycan link proteins (HAPLNs; predominantly HAPLN1 and 4) [14, 15]. Differential sulphation patterns of CSPGs can act also as binding sites for specific molecules, including axonal and neurite guidance cue semaphorin-3A (SEMA3A) [16].

PNNs have been linked to synaptic plasticity [17–19] and facilitation of fast-spiking activity [20, 21]. PNN formation is proposed to be activity dependent [22] and these structures play a significant role in cortical maturation. During development, PNNs increase in density and form ‘tighter’ nets around PVB interneurons. Incidentally, this ‘tightening’ of PNNs coincides with the closure of the critical period of cortical plasticity [23]. Essentially, it appears less dense PNNs infer a more permissive structural state which enables heightened synaptic plasticity [17, 24, 25]. As these nets condense, structural and functional plasticity is restricted [26]. Indeed, PNNs have been proposed to act as a molecular ‘brake’ which inhibits excessive plasticity and contributes to the closure of the critical period.

Transgenic models which knock out specific PNN components attenuate PNN formation, although the effect on PNN ablation appears to be dependent on the targeted component and, in some cases, is dose dependent. For example, conditional *Acan* knockout entirely ablates PNN formation [27], while mice heterozygous for *Acan* display anatomically normal PNNs [28]. Tenascin-R (TN-R) knockout mice present distinctly ‘fuzzy’ or granular PNNs which lack proximal dendrite coverage [28]. HAPLN1 knockout mice also show attenuated PNNs [29]. Interestingly, *Bcan* knockout mice do display Wisteria floribunda agglutinin (WFA) lectin-reactive PNNs (a widely used marker for PNNs) [28, 30]. These mice present long-term potentiation deficits, but show no overt deficits in learning or memory behaviours [31]. The intricacies of specific PNN component contribution to formation and function are yet to be fully understood. However, it is clear that CNS wide loss of PNN structures appears to produce functional consequences which can affect learning and memory behaviours, potentially due to their modulation of synaptic plasticity and axonal transduction [3, 27, 29, 30].

PNNs can be manipulated more locally via enzymatic digestion. One popular method of enzymatic digestion employs Chondroitinase ABC (ChABC; a bacterial enzyme derived from *Proteus vulgaris*). ChABC application in vitro and in vivo has proved successful in depleting PNNs, as assessed by the lack of WFA labelling. ChABC effects owe mainly to the enzyme’s ability to degrade sugar chains from CSPGs [32–34]. Only HAPLN1 remains bound to the cell surface following ChABC treatment [35].

Manipulation of PNNs by ChABC in the mature brain has gathered much attention due to reports which indicate that PNN digestion in vivo may be able to recapitulate a form of juvenile plasticity [24]. Additional work has shown that injection of ChABC to the primary visual cortex can facilitate structural and functional recovery of normal ocular dominance in rats which were subjected to a period of monocular deprivation [36]. ChABC appears to elevate the motility of cortical spines, and confers on them a higher degree of functional and structural plasticity [26]. Moreover, targeting ChABC to axons promotes neurite outgrowth and axonal spouting [37], further suggesting that PNN degradation can induce a state of heightened structural plasticity [38].

Considerable research efforts have focused on the modulation of synaptic plasticity after ChABC application, along with the effects on learning, memory and cognition [34, 39–42]. However, less is understood about the molecular phenotype induced. In particular, it is unclear how PNN attenuation via ChABC modulates the expression of genes which encode critical PNN components. To our knowledge, understanding how this manipulation impacts GABAergic and plasticity-related gene expression also remains ill-defined.

Here, we sought to define the molecular phenotype induced by ChABC digestion of PNNs. Moreover, we aimed to investigate whether ‘juvenile-like’ plasticity induces an immature molecular phenotype, and how this phenotype may differ from ‘classically’ induced mature homeostatic plasticity.

To this end, we treated primary cortical neuronal cultures with ChABC and measured expression of a variety of PNN-related, GABAergic, immediate early (IE) and developmentally regulated genes. We contrasted these with the results from primary neuronal cultures treated with known modulators of mature homeostatic plasticity, synaptic scaling and network activity; GABA_A_-receptor antagonist, bicuculline, and sodium channel blocker, tetrodotoxin (TTX).

The downstream effects of ChABC-induced PNN digestion on intracellular signalling have not been fully investigated. We have previously demonstrated modulation of PNN density and gene expression by brain-derived neurotrophic factor (BDNF) and c-Jun-NH_2_-terminal kinase (JNK) signalling. This work has shown that BDNF accelerates cortical interneuron maturation in vitro, and highlights that many BDNF effects on PNN development rely on appropriate JNK function and, potentially, upstream activator thousand-and-one amino acid kinase 2 (TAOK2). BDNF appears to be involved in the closure of the critical period [43–45] and is another known modulator of synaptic transmission and plasticity which has potent effects at adult synapses in multiple regions of the CNS [46].

Considering this, we investigated the effects of ChABC, bicuculline and TTX on MAPK signalling activity (via JNK and extracellular signal-regulated kinase (ERK) phosphorylation), along with expression of *Bdnf* and *Taok2*. Finally, we assessed whether TAOK signalling was involved in the effects of exogenous BDNF treatment on PNN-related, GABAergic and IE gene expression.

## Experimental Procedures

### Primary Neuronal Culture

Primary cortical neuronal cultures (which contain a mixture of neuronal cell types, including pyramidal cells and interneurons) were obtained from C57BL/6 mouse embryos at E17 and prepared according to our previously outlined procedures [47]. Briefly, pregnant mothers were euthanised (cervical dislocation) and embryos were removed immediately from the uterus. Cortices were dissected in ice-cold Hanks Balanced Salt Solution (HBSS), meninges removed and transferred into fresh HBSS. Cortical tissue was then finely homogenised, washed twice in HBSS and incubated in 0.05% trypsin/EDTA at 37ºC for 10 min. DMEM (supplemented with 10% HI horse serum, 1% penicillin–streptomycin, 1% Glutamax) was added to inactivate trypsin and tissues were subjected to centrifugation at 1500 rpm for 5 min at 4 °C. Following gentle trituration and resuspension, DMEM (9 ml/embryo) was added and neurons were seeded into polystyrene-based plastic plates which were pre-coated with 4 μg/ml poly-d-lysine and 6 μg/ml laminin. Neurobasal medium supplemented with B27 was added prior to cell seeding and allowed to equilibrate to incubator conditions for 30 min (37ºC, 5% CO_2_). Cells were seeded at a 50% dilution. Half-volume media changes with Neurobasal/B27 were performed at 24 and 48 h following seeding. Subsequently, 50% medium changes with Neurobasal/B27 were made every 3–4 days. For the present work, cells were allowed to mature for at least 14 days in vitro (DIV) prior to enzymatic or pharmacological treatment.

### Culture Treatments

For enzymatic degradation of PNNs, cultured cortical neurons were treated with small volumes of either vehicle (50 mM Tris–HCl/60 mM sodium acetate/0.02% BSA, pH 8.0) or ChABC (30 mU/ml) at 18 DIV for 3 DIV (treatment terminated at 21 DIV). Optimal ChABC concentration and treatment length were determined with reference to previous reports of in vitro use of the enzyme [22, 48]; suitable concentration confirmed via our own concentration gradient experiments (10, 30 and 100 mU/ml; Fig. S1).

To assess effects of the GABA_A_ receptor antagonist bicuculline [49] and the sodium channel blocker tetrodotoxin (TTX) [50] on gene and protein expression, cortical cultures were treated with either vehicle (dH_2_O/citrate buffer (0.1 M citric acid monohydrate/0.1 M tri-sodium citrate, pH 4.8)), 50 µM bicuculline [49, 51–53] (dH_2_0) or 2 µM TTX [54] (citrate buffer, 0.1 M citric acid monohydrate/0.1 M tri-sodium citrate, pH 4.8) for 16 h [55, 56] at 20 DIV (treatment terminated at 21 DIV).

To investigate the consequence of BDNF on PNN, GABAergic and IE gene expression, along with the reliance of any effects on TAOK, cells were treated with either vehicle (dH_2_0/DMSO), 30 µM CP-43 [57, 58] (TAOK inhibitor in DMSO), 50 ng/ml BDNF [59] (dH_2_0) or CP-43 + BDNF (30 µM and 50 ng/ml, respectively) at 14 DIV for 7 days (treatment terminated at 21 DIV).

### Immunofluorescent Labelling

Subsequent to ChABC-induced PNN degradation experiments, cells were fixed with 4% paraformaldehyde for 30 min on ice. Cells were permeabilised and blocked with PBS (0.3 M NaCl)/0.5%Triton X-100/10% normal goat serum (NGS) for 1 h at room temperature. Following this, cells were incubated overnight in a humidified chamber with primary antibodies diluted in 0.3 M PBS/0.5%Triton X-100/3% NGS at 4 °C. Cells were double labelled with anti-somatostatin (SST, ab183855, Abcam, 1:250) and biotinylated *Wisteria floribunda agglutinin* (WFA, B-1355–2, Vector Laboratories; 1:2000) lectin, which labels PNNs via preferential binding of glycans containing terminal N-acetylgalactosamine β1 residues on the chondroitin sulphate chains which compose these nets [6, 60]. Following primary antibody incubation, cells were washed three times (0.3 M PBS, 5 min/wash). Cells were incubated in appropriate secondary antibodies diluted in 0.3 M PBS/3% NGS for 1 h in the dark; for anti-SST, Alexa488-anti-rabbit (1:300; Jackson ImmunoResearch) and for WFA, streptavidin-conjugated Rhodamine Red-X (1:500; Jackson ImmunoResearch). Cells were washed a final three times before Vectashield mounting (Vector Laboratories, H-1200).

Prepared samples were visualised with a × 40 oil-immersion lens (Nikon). Confocal microscopy (BioRad, MRC 1024) was used to obtain Z-stacks (scanning 8-µm planes, 0.5-µm intervals). All settings were kept constant across related images. Projection images (summed z-stacks) were produced and used for subsequent qualitative analysis and representative images.

### RNA Isolation, cDNA Synthesis and Reverse Transcription–Quantitative PCR (RT-qPCR)

RNA isolation was performed using RNeasy mini-kits according to the manufacturers’ protocol (Qiagen, 74,104). RNA concentrations were normalised and cDNA was synthesised using high-capacity RNA to cDNA kit (ThermoFisher Scientific, 43,874,060) (including corresponding noRT condition for each synthesis reaction).

RT-qPCR primers were designed using NCBI primer blast in combination with Ensembl and obtained from Sigma. Sequences for all primer pairs are in contained Table S1. All primers were validated for product size and specificity via gel electrophoresis and visualisation. All primers were amplified within 90–110% efficiency (Fig. S2). Primers were diluted to 10 μM prior to use and RT-qPCR was performed using Fast SYBR Green Master Mix (Applied Biosystems, 4,309,155) in 20-μl reactions (ABI Prism). Cycle times—step 1: 50 °C, 2 min (1 cycle); step 2: 95 °C, 2 min (1 cycle); step 3: 95 °C, 10 s, 60 °C, 30 s (40 cycles).

Relative expression of each target gene was calculated via ΔΔCt method using *Gapdh* or *Tbp* as a housekeeping gene, selected based on stable expression across all experimental conditions. Housekeeping genes used for specific experiments can be found in corresponding figure legends.

### Protein Extraction

Protein from primary cortical neuronal cultures was extracted in line with our previously published protocols [61]. In brief, culture medium was removed, plates were placed on ice and 1000 µl of ice-cold PBS (pH 7.5) was added for 15–30 s. Upon wash buffer removal, 80 µl of RIPA buffer (10 nM Tris–HCl, 150 mM NaCl, 1 mM EGTA, 0.5% NP40, 0.1% SDS, 0.1% sodium deoxycholate) was added. Cells were lysed briefly, after which wells were manually scraped (~ 30 s/well) and contents were removed within 10 min. Extracts were centrifuged at 13,000 rpm for 10 min at 4 °C. Subsequently, supernatants were collected and protein concentration determined via Bradford protein assay [62]. Samples were stored at − 80 °C for downstream analysis.

### Protein Electrophoresis and Western Blot

For each sample, 50 µg of protein was prepared with 4 × sample buffer (NuPAGE, Novex, NP0007) and sample reducing agent (NuPAGE, Novex, NP0004) prior to denaturation (80 °C for 10 min). Samples were then subjected to SDS-PAGE in 10% Bis–Tris gels (120 V, 2 h) and transferred to PVDF membranes (30 V, 1 h). Sample loading order was alternated across individual gels to ensure conditions were not systematically run in the same location of the gel. Following transfer, membranes were washed twice in dH_2_O and blocked for 30 min (0.5% Tween–Tris-buffered saline (TTBS) + 3% dried milk). Membranes were incubated in primary antibodies overnight at 4 °C with agitation (pJNK: Abcam, ab76572, 1:12,000; pERK; CST, #4377, 1:5000, diluted in SignalBoost Immunoreaction Enhancer solution (Millipore, 407,207)). Following incubation, membranes were washed (3 × TTBS, 10 min) and incubated in HRP-conjugated anti-rabbit secondary antibody (1:10,000, Sigma-Aldrich, 12–348) for 2 h. Membranes were washed again (once TTBS, twice 10 × TBS) prior to imaging. Blots were further probed with HRP-conjugated GAPDH antibody (1:20,000, Genetex, GTX627408-01). Membrane-bound antibodies were detected via a chemiluminescent HRP substrate (Immobilon, Millipore, WBKLS0100), and digital images were captured via PXi4 (Syngene).

Quantification of digital images was performed in ImageJ, with band intensity values measured and normalised to GAPDH. Normalised data were expressed as a percentage of vehicle mean.

### Data Analysis

Data were analysed via one-way ANOVA with Tukey post hoc multiple comparisons or, when required, Mann–Whitney *U* tests (Minitab 17). Results were considered significant if *p* < 0.05. Details of specific analysis for particular experiments can be found in corresponding figure legends. A complete table of statistics (*F* and *p* values) can be found in Table S2. Boxplots and heatmaps were produced in Prism 9.

## Results

### ChABC-Induced PNN Degradation Dysregulated Key PNN-Related, GABAergic and Immediate Early (IE) Gene Expression

**Fig. 1:**
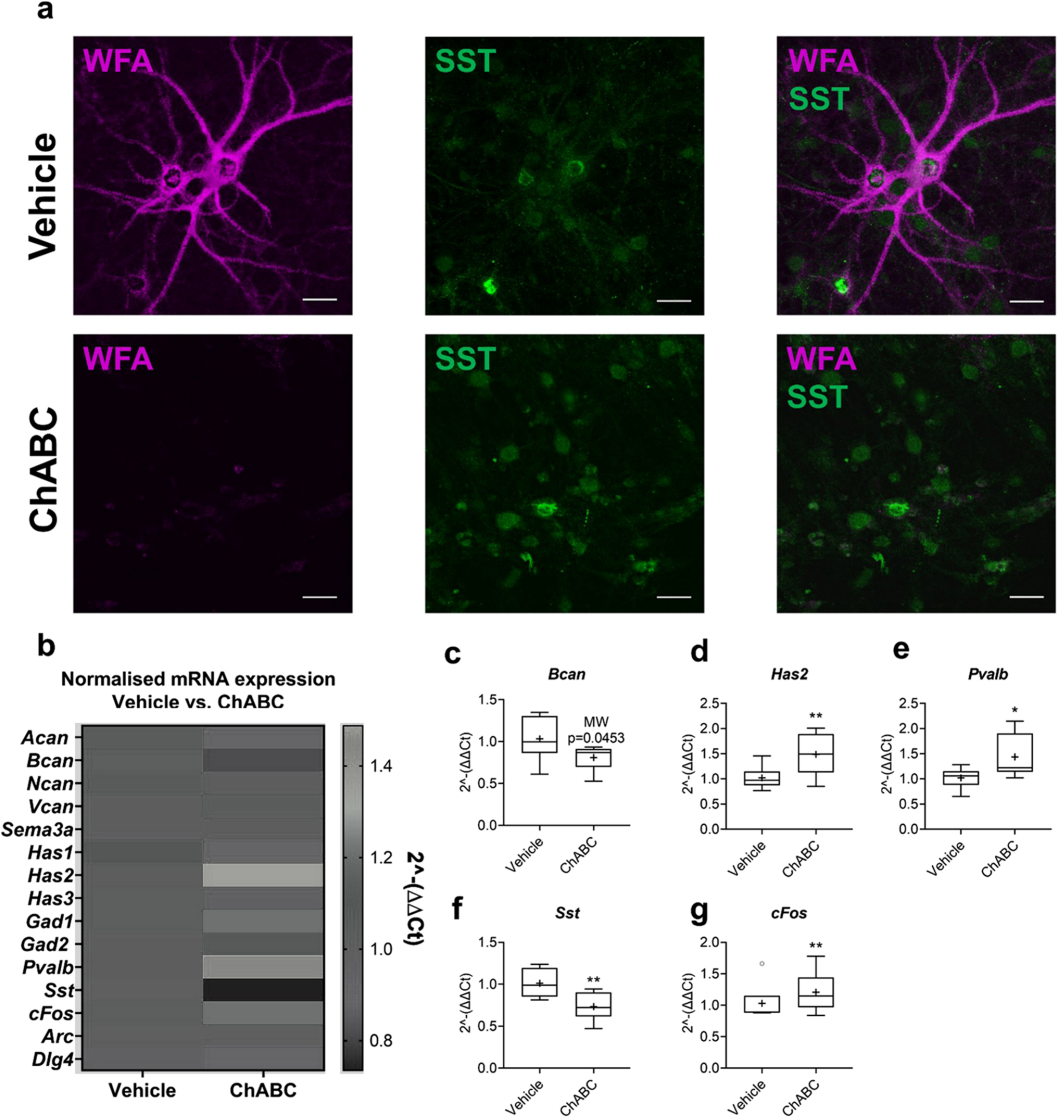
In vitro ChABC degradation of PNNs dysregulated GABAergic and PNN-related gene expression, while upregulating *cFos* expression. **a** Representative images of primary cortical neuronal cultures treated with either vehicle or ChABC (30 mU/ml) from 18 to 21 DIV. Cultures were labelled with anti-SST (green) and WFA (magenta). A distinct lack of PNN structures was qualitatively observed in cultured treated with ChABC. *N* = 5 independent cultures. Scale bars = 30 µm. Expression of key PNN-related, GABAergic and immediate-early genes were measured via RT-qPCR. **b** Heatmap of mean normalised mRNA expression of neurons treated with either vehicle or ChABC from 18 to 21 DIV (30 mU). RNA was isolated and cDNA synthesised at 21 DIV. Only genes showing a statistically significant change in expression are further presented as boxplots. **c, d** Compared to vehicle, ChABC downregulated *Bcan* expression (Mann–Whitney: CI(0.013, 0.48), *p* = 0.045), while *Has2* was significantly upregulated (*p* = 0.038). **e, f** An opposing change was found in *Pvalb* and *Sst* in response to ChABC treatment, with the upregulation of *Pvalb* (*p* = 0.044) and downregulation of *Sst* (*p* = 0.019). **g** A moderate increase in *cFos* expression was observed (*p* = 0.043). There were no significant changes in expression of other CSPGs or PNN-related genes. *Gad1*, *Gad2*, *Dlg4* and *Arc* expression were also not significantly affected by ChABC treatment. Relative expression was calculated using ∆∆Ct method with *Gapdh* housekeeping gene. *N* = 10 independent samples derived from 3 independent cultures. Data are presented as boxplots with medians, interquartile ranges and ‘Tukey’ whiskers; crosses indicate sample means. Data were analysed via one-way ANOVA with Tukey post hoc multiple comparisons. * and ** represent Tukey post hoc significance compared to vehicle (*p* < 0.05 and *p* < 0.01, respectively). In addition, a Mann–Whitney *U* test was performed for vehicle vs. ChABC on *Bcan*

Our initial experiments sought to understand the consequences of ChABC-induced PNN degradation on expression of key PNN, GABAergic and immediate early (IE) genes. Using double immunofluorescent labelling with WFA lectin and anti-SST, we confirmed successful PNN ablation in primary neuronal cultures treated at 18 DIV with either vehicle or ChABC (30 mU/ml) for 3 DIV (Fig. 1a). As aforementioned, while PNNs primarily enwrap PVB-expressing interneurons, these structures have been shown to localise around a small subset of SST-expressing interneurons which express voltage-gated potassium channel subunit Kv3 [7, 63]. Technical limitations encountered with PVB immunofluorescent labelling in vitro led us to co-label with SST. Our qualitative assessment of cultures revealed clear WFA + /SST + and WFA + /SST − labelling in vehicle-treated cultures; no WFA + /SST + or WFA + /SST − was found in any ChABC-treated cultures, indicating successful enzymatic digestion of PNNs (Fig. 1a and Fig. S1).

RT-qPCR analysis of gene expression revealed a distinct downregulation of *Bcan* (Fig. 1c), a CSPG which is prevalent throughout the mature PNN structure but is thought to be enriched around the axonal initial segment [64, 65], after ChABC treatment. We found no significant changes in any other CSPG components of PNNs (Fig. 1b). However, compared to vehicle, membrane-bound *Has2* which is one of three enzymes (HAS1–3) that synthesise the hyaluronan backbone upon which CSPGs bind, was upregulated after ChABC treatment (Fig. 1d).

Our results also indicated dysregulation to two dominant and distinct interneuron subtypes following ChABC treatment. RT-qPCR revealed a significant upregulation in *Pvalb* expression and simultaneous downregulation of *Sst* in cultures treated with ChABC, compared to vehicle (Fig. 1e, f). In addition, our data indicate a substantial upregulation in *cFos* (Fig. 1g), an IE gene which, while not a specific marker for GABAergic interneurons, is widely used as a functional marker of neuronal activity [66].

### GABAA Antagonist, Bicuculline, Upregulated Acan and Immediate Early (IE) Gene Expression

Our data indicate a dysregulation to expression of key PNN components (*Bcan* and *Has2*, both of which are developmentally regulated and have been implicated in synaptic plasticity and stability [31, 67–69]), *cFos*, *Pvalb* and *Sst*. These changes indicate a potential dysregulation of inhibitory interneuron dynamic, network activity and, potentially, synaptic plasticity. Our following experiments investigated consequences of two known modulators of excitatory–inhibitory balance, network activity, synaptic scaling and plasticity: GABA_A_ antagonist bicuculline and sodium channel blocker tetrodotoxin (TTX). To this end, primary cortical neuronal cultures were treated with either vehicle, bicuculline (50 µM) or TTX (2 µM) for 16 h prior to RNA isolation at 21 DIV.

**Fig. 2:**
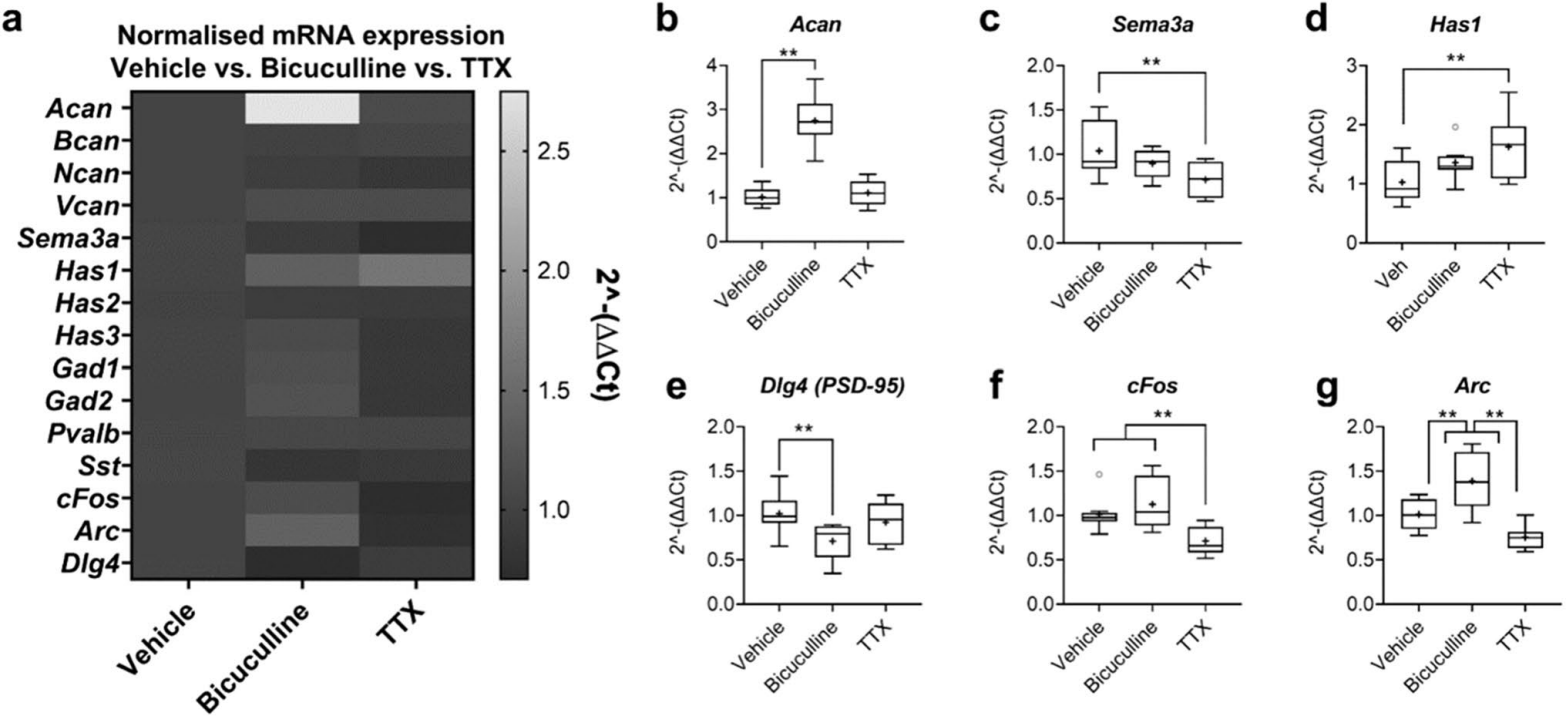
Bicuculline increased IE gene expression and upregulated expression of *Acan.* Primary cortical neuronal cultures were treated with vehicle, bicuculline (50 µM) or TTX (2 µM) at 20 DIV for 16 h. RNA was isolated at 21 DIV, cDNA synthesised and gene expression analysed via RT-qPCR. Heatmap of mean expression of normalised mRNA expression of key PNN-related, GABAergic and IE genes (**a**). Only genes showing a statistically significant change in expression are further presented as boxplots. Analysis revealed a substantial increase in *Acan* expression after bicuculline (main effect of treatment, *F*_2,21_ = 52.59, *p* < 0.000; Tukey, vehicle vs. bicuculline: *p* < 0.01) (**b**). Expression of all other CSPG genes were unaffected by bicuculline or TTX. In addition, TTX treatment significantly downregulated expression of *Sema3a* (main effect of treatment, *F*_2,21_ = 3.82, *p* = 0.038; Tukey, vehicle vs. TTX: *p* < 0.01) (**c**) and upregulated *Has1* (main effect of treatment, *F*_2,21_ = 4.35, *p* = 0.026; Tukey, vehicle vs. TTX: *p* < 0.01) (**d**). In terms of GABAergic gene expression, there were no significant differences in *Pvalb*, *Sst*, *Gad1* or *Gad2* compared to vehicle, after either bicuculline or TTX. However, bicuculline downregulated *Dlg4* (PSD-95) (**e**). Assessment of activity-dependent markers revealed elevated expression of IE genes, *cFos* and *Arc*, after bicuculline (main effect of treatment, *F*_2,21_ = 7.58, *p* = 0.003; Tukey, vehicle vs. bicuculline: *cFos: p* < 0.01, *Arc*: *p* < 0.01). However, compared to vehicle, TTX reduced *cFos* expression (Tukey, vehicle vs. TTX: *p* < 0.01). *Arc* was significantly lower in TTX vs. bicuculline conditions (Tukey, bicuculline vs. TTX: *p* < 0.01) (**f, g**)*.* Relative expression was calculated using ∆∆Ct method with *Tbp* housekeeping gene. *N* = 8 independent samples derived from 4 independent cultures per condition. Data are presented as boxplots with medians, interquartile ranges and ‘Tukey’ whiskers; crosses indicate sample means. Data were analysed via one-way ANOVA with Tukey post hoc multiple comparisons. ** represents Tukey post hoc significance compared to vehicle (*p* < 0.01)

RT-qPCR revealed a clear upregulation of *Acan* in response bicuculline, but not TTX, treatment (Fig. 2b). In addition, compared to vehicle, bicuculline substantially increased expression of both *Arc* and *cFos*, whereas *cFos* expression was reduced in TTX-treated cultures (Fig. 2f, g). *Arc* expression was also significantly lower in TTX-treated cultures compared to those treated with bicuculline (Fig. 2f). Our data also showed a significant downregulation of *Dlg4* (PSD-95) in bicuculline-treated cultures compared to vehicle (Fig. 2e).

Interestingly, TTX treatment demonstrated no apparent effect on CSPG expression (Fig. 2a). However, TTX-treated cultures expressed significantly lower *Sema3a* (encoding the secreted chemorepulsive protein SEMA3A which binds to PNNs via interactions with chondroitin sulphate). A modest increase in *Has1* mRNA expression was also found (Fig. 2c, d).

### ChABC Treatment Decreases JNK Phosphorylation

The role of JNK signalling in several developmental processes within the CNS has become apparent [70–75]. Our previous work has highlighted JNK and other MAPK signalling molecules, including TAOK2 and ERK, as key players in the maturation of GABAergic interneurons [76]. More specifically, our work has revealed a modulatory role for JNK in the development and regulation of PNNs. However, as yet, the effects of PNN ablation on neuronal MAPK signalling remain unclear. Hence, we aimed to understand the consequences of enzymatic PNN digestion on two key MAPK signalling pathways, JNK and ERK.

**Fig. 3:**
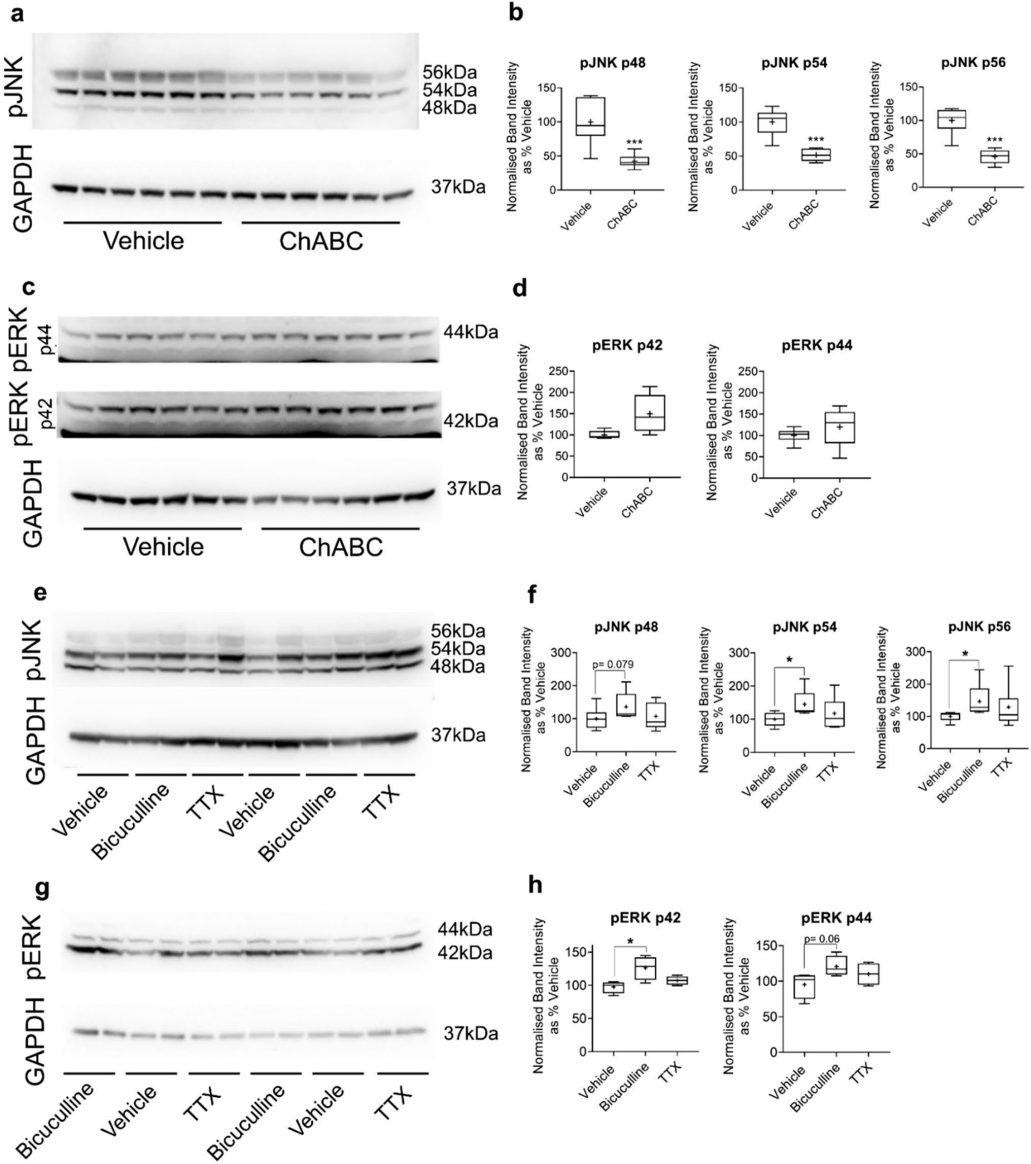
ChABC and bicuculline differentially regulated MAPK signalling pathways. pJNK and pERK levels were assessed in protein extracts from primary cortical neuronal cultures via Western blots. Cultures were treated with either vehicle vs. ChABC (30 mU/ml; 18–21 DIV) (**a**–**d**; *N* = 6 independent samples derived from 3 independent cultures per condition) or vehicle, bicuculline (50 µM) or TTX (2 µM) (20–21 DIV, 16-h stimulation) (**e**–**h**; *N* = 8 independent samples derived from 4 independent cultures per condition). ChABC degradation of PNNs substantially reduced levels of all pJNK isoforms (*p* < 0.001) (**a, b**). pERK levels (**c, d**) were not significantly changed by ChABC treatment. Bicuculline upregulated levels of pJNK p54 and p56 isoforms (*p* < 0.05) and data suggested a possible trend towards bicuculline upregulation of pJNK p48 (*p* = 0.079). Bicuculline increased pERK p42 (*p* < 0.05), with a trend towards increased pERK p44 (*p* = 0.06). TTX treatment did not significantly alter pJNK or pERK levels (**e, f**). Band intensity was measured via ImageJ and normalised to GAPDH. Intensity values were then expressed as a percentage of vehicle. Data are presented as boxplots with medians, interquartile ranges and ‘Tukey’ whiskers; crosses indicate sample means. Data were analysed via one-way ANOVA with Tukey post hoc multiple comparisons, *** represents *p* < 0.001 compared to vehicle

Western blot quantification revealed a distinct reduction in all phosphorylated JNK (pJNK) isoforms (p48, p54 and p56) in cultured primary cortical interneurons treated with ChABC, compared to vehicle (Fig. 3a, b). Our data indicate no clear difference in levels of phosphorylated ERK (pERK) isoforms (p42 or p44) in cultured cortical neurons treated with ChABC, compared to vehicle (Fig. 3c, d).

Given the suggested role of PNNs in synaptic plasticity, we contrasted the above results of ChABC treatment on pJNK and pERK levels with the consequences of bicuculline and TTX administration on activity within these pathways. Western blots revealed an increase in pJNK p54 and p56 after bicuculline, compared to vehicle (Fig. 3e, f). There was also a non-significant, but suggestive, trend towards an increase in pJNK p48 following bicuculline treatment (*p* = 0.079). No tangible differences in pJNK expression were found in response to TTX treatment. Similarly, there were no significant differences in levels of pERK after TTX administration in cultured cortical neurons. However, bicuculline appeared to increase pERK (pERK p42, *p* < 0.05; pERK p44, *p* = 0.06).

### ChABC-Induced PNN Degradation Induces an Immature Neuronal Phenotype In Vitro

Our data indicate clear differences between the effects of ChABC and bicuculline/TTX treatment in cortical neuronal cultures. In particular, a number of markers which are related to synaptic plasticity, network activity and interneuron network dynamics (including *Arc*, *Pvalb*, *Sst, Dlg4* expression, along with pJNK and pERK activity) are differentially regulated by these treatments. Moreover, our data indicate that ChABC, bicuculline and TTX treatments produce divergent expression patterns of PNN components.

As previously discussed, the maturation of PNNs may act as so-called brakes on plasticity, with the maturation of these nets being linked with closure of the critical period and the associated state of ‘juvenile’ plasticity. Considering this, we sought to understand whether the observed phenotypic differences in ChABC versus bicuculline/TTX treatment indicated the initiation of a ‘juvenile-like’ phenotype by ChABC-induced PNN ablation, rather than a more classical modulation of plasticity within mature cortical neurons. Accordingly, we measured expression of four developmentally regulated genes (*Ascl1, Dlx2* and *Pax6,* transcription factors important in early cortical development*,* and *Kcnc1*, a potassium channel subunit expressed later in development) in cultured primary cortical neurons treated with either vehicle, ChABC, bicuculline or TTX.

**Fig. 4:**
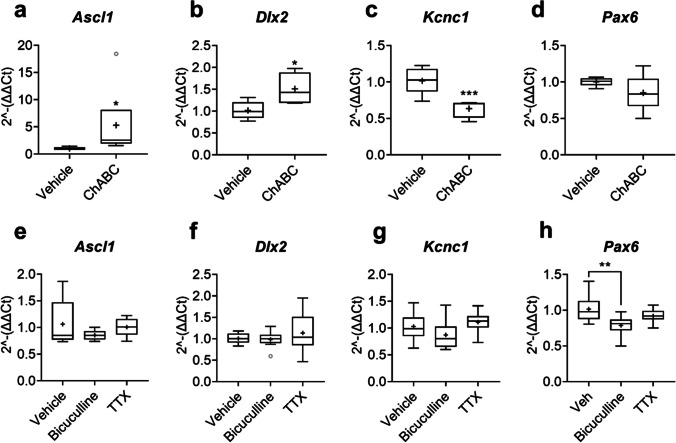
ChABC treatment produced a pattern of gene expression indicative of immature interneurons. Expression of developmentally regulated genes, *Ascl1*, *Dlx2*, *Kcnc1* and *Pax6*, were assessed in primary cortical neuronal cultures treated with either vehicle or ChABC (30 mU/ml) from 18 to 21 DIV (**a–d**) or vehicle, bicuculline (50 µM) or TTX (2 µM) from 20 to 21 DIV (16-h stimulation) (**e–h**) via RT-qPCR. The expression of these genes was interpreted as a measurement of relative maturity of neuronal cultures, with *Dlx2* and *Kcnc1* predominantly reflecting interneuron maturity status. We found substantial increases in *Dlx2* (*p* = 0.012) and *Ascl1* (MASH1) (*p* = 0.050), along with a significant decrease in *Kcnc1* expression (*p* = 0.001), after ChABC treatment. No differences were observed in *Dlx2, Ascl1* or *Kcnc1* expression after bicuculline or TTX stimulation. Bicuculline treatment decreased *Pax6* expression (main effect of treatment, *F*_2,21_ = 4.71, *p* = 0.020; Tukey, vehicle vs. bicuculline: *p* < 0.01), whereas no significant difference was found in *Pax6* expression after ChABC treatment. Relative expression was calculated using ∆∆Ct method with *Gapdh* housekeeping gene. *N* = 8 independent samples derived from 4 independent cultures per condition. Data are presented as boxplots with medians, interquartile ranges and ‘Tukey’ whiskers; crosses indicate sample means. Data were analysed via one-way ANOVA with Tukey post hoc multiple comparisons. *, ** and *** represent Tukey post hoc significance compared to vehicle (*p* < 0.05, *p* < 0.01 and *p* < 0.001, respectively)

We found upregulated expression of both *Ascl1* (otherwise known as MASH-1) and *Dlx2* after ChABC PNN digestion, compared to vehicle (Fig. 4a, b). In addition, compared to vehicle, ChABC treatment downregulated expression of developmentally regulated *Kcnc1* (Fig. 4c), a gene which encodes Kv3.1b potassium channels that are enriched in fast-spiking PVB interneurons [77, 78]. No difference in expression of the transcription factor *Pax6* was found in response to ChABC treatment.

RT-qPCR revealed no differences in *Ascl1*, *Dlx2* or *Kcnc1* expression after either bicuculline or TTX treatment, compared to vehicle. However, a modest downregulation of *Pax6* was found after bicuculline administration.

### ChABC Upregulated Taok2 Expression with No Effect on Bdnf mRNA Levels

**Fig. 5:**
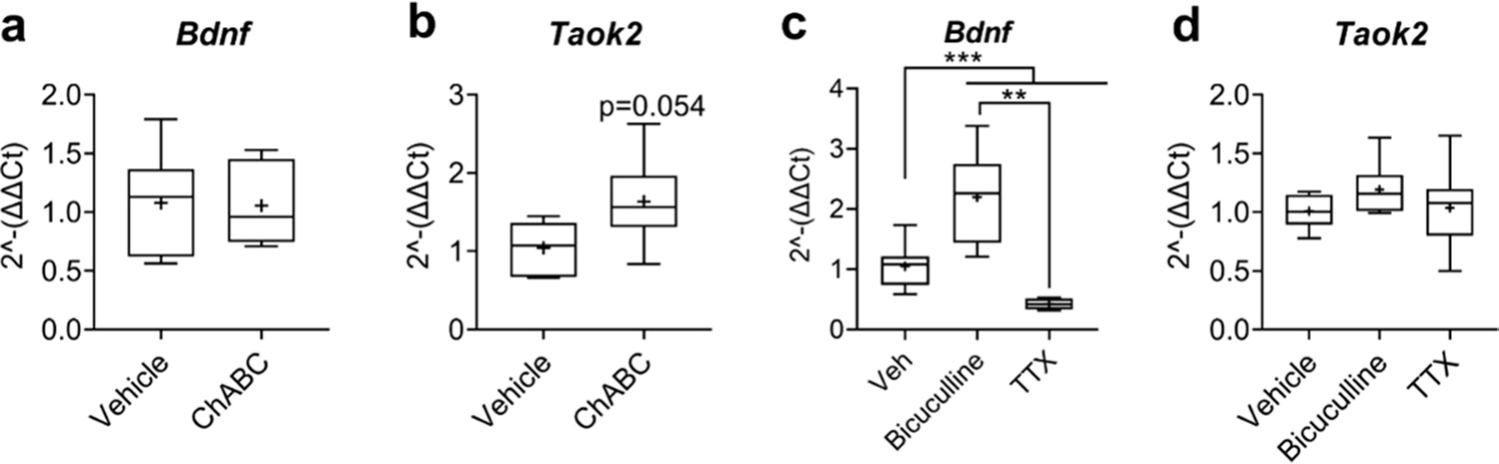
*Taok2* expression was elevated after ChABC with no effect on *Bdnf* expression, while both bicuculline and TTX modulated *Bdnf* expression with no significant consequence to *Taok2* expression. Expression of *Bdnf* and *Taok2* were measured via RT-qPCR in cultures treated with either (**a, b**) vehicle or ChABC (30 mU/ml) from 18 to 21 DIV, or (**c, d**) vehicle, bicuculline (50 µM) or TTX (2 µM) from 20 to 21 DIV (16-h stimulation). ChABC treatment had no effect on *Bdnf* expression, whereas bicuculline upregulated and TTX downregulated *Bdnf* (main effect of treatment, *F*_2,21_ = 28.25, *p* < 0.000; Tukey vehicle vs. bicuculline: *p* < 0.001, vehicle vs. TTX: *p* < 0.001, bicuculline vs. TTX: *p* < 0.01). Conversely, ChABC increased *Taok2* expression (*p* = 0.054), while both bicuculline and TTX had no effect. Relative expression was calculated using ∆∆Ct method with *Gapdh* housekeeping gene. *N* = 8 independent samples derived from 4 independent cultures per condition. Data are presented as boxplots with medians, interquartile ranges and ‘Tukey’ whiskers; crosses indicate sample means. Data were analysed via one-way ANOVA with Tukey post hoc multiple comparisons. ** and *** represent Tukey post hoc significance compared to vehicle (*p* < 0.01 and *p* < 0.001, respectively)

Adequate BDNF expression is crucial to the closure of the critical period of cortical plasticity [43–45]. Our data regarding expression of developmentally regulated genes (Fig. 5) indicated a potential induction of a ‘juvenile’-like state of cultured cortical neurons which may be more akin to a ‘re-opening’ of the critical period of cortical plasticity, rather than a heightened state of mature synaptic plasticity induced by bicuculline. With this in mind, we measured *Bdnf* expression in cultures treated with either vehicle, ChABC, bicuculline or TTX. In addition, given the clear impact of ChABC-induced PNN digestion on pJNK, we investigated whether expression of upstream kinase *Taok2* might be altered in these cultures.

RT-qPCR analysis revealed no difference in *Bdnf* expression after ChABC treatment, compared to vehicle (Fig. 5a). Heightened levels of *Taok2* mRNA were observed in neuronal cultures treated with ChABC (Fig. 5b). Conversely, we observed heightened *Bdnf* mRNA expression after bicuculline treatment (Fig. 5c), with no effect on *Taok2* expression (Fig. 5d). TTX treatment downregulated *Bdnf* expression but, similarly to bicuculline, produced no change in *Taok2* mRNA levels (Fig. 5c, d).

### BDNF Treatment Upregulated Expression of Key PNN Components, Increased Gad Expression and Elevated IE Gene Expression in a TAOK-Dependent Manner

BDNF is another known modulator of synaptic transmission and plasticity which has potent effects at adult synapses. Our previous work has highlighted that BDNF treatment of immature primary cortical neuronal cultures accelerates cortical interneuron maturation, and in particular, increases PNN density in a JNK-dependent manner [76]. This work has also implicated TAOK2, an upstream activator of JNK, in the appropriate development of PVB + interneurons and PNNs.

Considering this, we investigated the effects of exogenous BDNF treatment from 14 to 21 DIV on PNN-related and GABAergic gene expression and aimed to elucidate whether any effects were reliant on TAOK. We measured PNN, GABAergic and IE gene expression in cultures treated with either vehicle, TAOK inhibitor (CP-43), BDNF or CP-43 + BDNF.

**Fig. 6:**
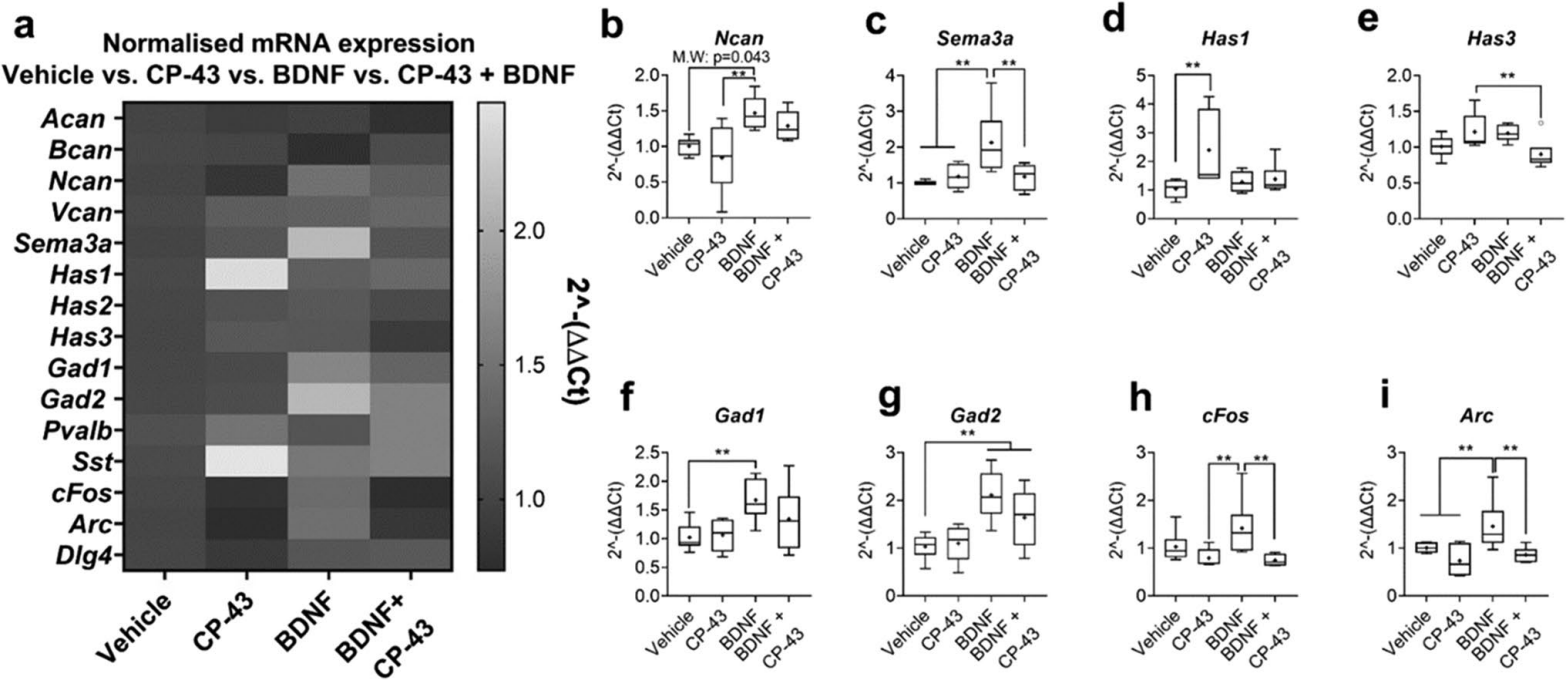
BDNF treatment upregulated expression of key PNN components, increased *Gad1/Gad2* and elevated IE gene expression in a TAOK-dependent manner. Primary cortical neuronal cultures were treated with either vehicle, TAOK inhibitor (CP-43; 30 μM), BDNF (50 ng/ml) or CP-43 + BDNF from 14 to 21 DIV. Expression of key PNN-related, GABAergic and IE genes were measured via RT-qPCR. Heatmap of mean expression of normalised mRNA expression of key PNN-related, GABAergic and immediate-early genes (**a**). Only genes showing a statistically significant change in expression are further presented as boxplots. We found that BDNF significantly increased *Ncan* (Mann–Whitney: CI (0.0380, 0.7895), *p* = 0.043) (**b**) and *Sema3a* (main effect of treatment, *F*_3,19_ = 5.60, *p* = 0.006; Tukey, vehicle vs. BDNF: *p* < 0.01) (**c**); this effect was attenuated by TAOK inhibition (Tukey, BDNF vs. CP-43 + BDNF: *p* < 0.01). TAOK inhibition alone significantly upregulated expression of *Has1* (main effect of treatment, *F*_3,19_ = 3.63, *p* = 0.032; Tukey, vehicle vs. CP-43: *p* < 0.01) and there was a non-significant trend towards CP-43 increasing *Has3* expression (**d, e**). These CP-43-dependent elevations appear to be attenuated with the application of exogenous BDNF (i.e. CP-43 vs. BDNF + CP-43; *Has3*: Tukey, CP-43 vs. BDNF + CP-43, *p* < 0.01). BDNF upregulated expression of *Gad1* (main effect of treatment, *F*_3,19_ = 3.56, *p* = 0.034; Tukey, vehicle vs. BDNF: *p* < 0.01) and *Gad2* (main effect of treatment, *F*_3,19_ = 7.29, *p* = 0.002; Tukey, vehicle vs. BDNF: *p* < 0.01) (**f, g**). There was a non-significant trend towards TAOK inhibition attenuating this effect. BDNF treatment also elevated expression of IE genes, *cFos* (main effect of treatment, *F*_3,19_ = 4.13, *p* = 0.021; Tukey, vehicle vs. BDNF: *p* < 0.01) and *Arc* (main effect of treatment, *F*_3,19_ = 4.05, *p* = 0.022; Tukey, vehicle vs. BDNF: *p* < 0.01) (**h, i**). Again, this elevation was negated when TAOK was inhibited (Tukey, BDNF vs. BDNF + CP-43: *cFos: p* < 0.01, *Arc: p* < 0.01). Relative expression was calculated using ∆∆Ct method with *Gapdh* housekeeping gene. *N* = 8 independent samples derived from 4 independent cultures per condition. Data are presented as boxplots with medians, interquartile ranges and ‘Tukey’ whiskers; crosses indicate sample means. Data were analysed via two-way ANOVA with Tukey post hoc multiple comparisons. Factors BDNF and inhibitor, with BDNF and inhibitor crossed to investigate any potential interaction. ** represents Tukey post hoc significance compared to indicate condition (*p* < 0.01). In addition, a Mann–Whitney *U* test was performed for vehicle vs. BDNF on *Ncan* data

Our data revealed a clear BDNF-induced elevation of *Ncan*, *Sema3a*, *Gad1*, *Gad2*, *cFos* and *Arc* (Fig. 6a–c, f–i). In the case of *Sema3a*, *cFos* and *Arc*, this upregulation was attenuated when CP-43 and BDNF were administered concomitantly. An observable reduction in *Ncan*, *Gad1* and *Gad2* expression was present in BDNF + CP-43 conditions, compared to BDNF-only, although the effects were not statistically significant.

TAOK inhibition via CP-43 upregulated *Has1* expression; this increase was not observed in CP-43 + BDNF conditions (Fig. 6d). A modest, although non-significant, elevation in *Has3* expression was also found after CP-43 treatment (Fig. 6e). Again, this heightened expression was attenuated when BDNF was administered alongside CP-43 (Fig. 6).

## Discussion

Our work aimed to understand the molecular consequences of ChABC digestion of PNNs, with a specific focus on expression of PNN-related, GABAergic and IE genes, and MAPK signalling. MAPK signalling downstream of BDNF has been linked with GABAergic interneuron maturation. Hence, we investigated the effects of ChABC treatment on key MAPK signalling pathways, JNK and ERK. We contrasted ChABC-induced changes with those observed after the application of well-known modulators of mature synaptic plasticity and network activity in cortical neurons, bicuculline and TTX. PNN digestion resulted in an immature molecular phenotype as evidenced by an upregulation of *Has2*, *Ascl1* and *Dlx2* together with downregulation of *Kcnc1* and *Bcan*. PNN ablation also downregulated JNK activity. Conversely, bicuculline and TTX did not induce a phenotype typical of immaturity, and bicuculline upregulated phosphorylation of several JNK and ERK isoforms. Finally, in light of work demonstrating JNK-dependent effects of BDNF on the regulation of interneuron and PNN development, we examined the involvement of upstream kinase, TAOK, on BDNF modulation of PNN and plasticity-related genes. We found that BDNF modulation of IE genes and PNN component, *Sema3a*, was TAOK-signalling dependent.

### In Vitro ChABC Digestion of PNNs Induces an Immature Molecular Phenotype in Cortical Neurons

Our data indicate the induction of an immature neuronal phenotype after enzymatic degradation of PNNs in vitro. Our initial experiments revealed that ChABC treatment of primary neuronal cultures downregulated *Bcan* expression, while it upregulated *Has2* mRNA levels. Previous work has highlighted a tight developmental regulation of these genes. *Bcan* expression is lowest during embryonic development and maximal in the mature brain, with upregulation becoming particularly prominent around the closure of the critical period of cortical plasticity [79]. Conversely, in vitro *Has2* expression is highest between 3 and 14 DIV, after which expression decreases to almost non-detectable levels at 21 DIV [69].

These primary indications of an immature neuronal phenotype after ChABC treatment were strengthened by assessment of well-characterised developmentally regulated genes, *Ascl1*, *Dlx2* and *Kcnc1*. We found upregulated expression of both *Ascl1* (otherwise known as MASH-1) and *Dlx2* after ChABC PNN digestion. *Ascl1* is best known for its role in neurogenesis and as a neuronal progenitor marker [80, 81]. Transcription factor, *Dlx2*, guides a number of developmental programmes which are critical to GABAergic development, including tangential interneuron migration to the neocortex and interneuron differentiation [82–84]. Both *Ascl1* and *Dlx2* are highly expressed during early developmental stages, with their expression diminishing to extremely low levels between postnatal day 10 and 15 [85]. In addition, ChABC treatment downregulated expression of *Kcnc1*, a gene which encodes Kv3.1b voltage-gated potassium channels that are enriched in fast-spiking PVB + interneurons [77, 78]. *Kcnc1* expression is almost undetectable throughout early development, with expression steadily increasing to maximal levels in the mature brain [85]. Previous work has shown that BCAN forms protein complexes with both Kv1.1 and Kv3.1b and has shown that *Bcan* knockdown reduces Kv3.1b density on PVB + interneurons (likely, of the basket cell subtype) [86]. The relationship between voltage-gated potassium channel Kv3.1b and BCAN adds further interest to our finding that both *Bcan* and *Kcnc1* are downregulated after PNN digestion.

No difference in expression of the transcription factor *Pax6* was detected in response to ChABC treatment. *Pax6* expression is tightly linked to glutamatergic cell differentiation, with maximal expression during embryonic and early postnatal development [87, 88]. After this period, expression of *Pax6* rapidly declines and is detectable only in immature neurons of the olfactory bulb and dentate gyrus where it may be associated with adult neurogenesis [89].

We suggest that this clear dysregulation of *Bcan*, *Has2*, *Dlx2* and *Kcnc1*, coupled with no alteration to glutamatergic associated *Pax6*, may illustrate an immature phenotype elicited by ChABC. This may specifically affect cortical interneurons. Indeed, this suggestion is in line with previous reports which appear to show predominant effects of ChABC on inhibitory interneuron physiology, with no impact on intrinsic excitatory cell properties [22, 90]. However, PNN constituents can also be found at synapses on excitatory neurons [91], and the changes in Arc observed are likely to reflect changes in activity of principal cells, so this hypothesis requires further investigation.

### Upregulation of Pvalb Expression Contrasts with Reversion to Immature Phenotype

We found an upregulation of *Pvalb* following ChABC digestion of PNNs. Previous work has demonstrated that induction of *Pvalb* mRNA expression correlates with the functional maturation of cortical interneurons [92, 93]. In fact, there is evidence that PVB interneuron maturation is responsible for critical period closure [27, 94]. This finding may appear counterintuitive to our data which suggest an immature interneuron phenotype in vitro. However, some previous work has highlighted that attenuation of PNNs via ChABC selectively increases the excitability of fast-spiking (FS) PVB interneurons [22], although there are conflicting reports [20]. Nonetheless, given that *Pvalb* expression appears to be activity dependent [95–97], an elevation in *Pvalb* expression after ChABC could be in line with previous observations of heightened PVB cell excitability following ChABC treatment in vitro.

Our findings of increased *Pvalb* and *Ascl1* mimic reports from tenascin-R deficient mice, in which PVB cell number and *Ascl1* expression are increased [98]. Tenascin-R is an extracellular link protein which contributes to the assembly and stabilisation of PNNs, and mice lacking tenascin-R have severely compromised PNNs [99]. Hence, it is interesting that the chronic impairment of PNNs in these animals produces a somewhat similar result to the data we report from ChABC PNN digestion in vitro.

The data presented in the current work are insufficient to provide conclusive evidence regarding the functional consequence of a ChABC-induced immature molecular phenotype on PVB interneuron spiking activity or wider functional consequences to the inhibitory interneuron network. However, they do demonstrate regulation of mRNAs related to key subclasses of cortical GABAergic interneurons: ChABC digestion of PNNs upregulated *Pvalb* and downregulated *Sst* expression. Together, *Pvalb* and *Sst* interneuron subclasses form a major control mechanism which set cortical excitatory–inhibitory balance [100–102]. PVB interneurons exert strong inhibition onto pyramidal cells, but also inhibit other PVB interneurons. In addition, SST interneurons inhibit all other interneuron subclasses, but not one another [103]. ChABC-induced changes to these markers could indicate changes to inhibitory network dynamics following PNN degradation.

Our data which demonstrate a ChABC-induced reduction of *Kcnc1*, indicating a loss of Kv3.1b channels, which endow PVB interneurons with their FS ability [104–107], may support the notion of altered electrophysiological characteristics of interneurons following PNN removal. Future work would be well placed to investigate the functional consequences of a ChABC-induced immature molecular phenotype. This work may provide a richer understanding of the cellular phenotype underpinning the apparent juvenile-like plasticity which has previously been reported by in vivo ChABC studies [24, 37].

It is important to note that no significant change in Gad1 expression was found in ChABC-treated cultures (Fig. 1a). However, it is possible that the opposing regulation of two distinct interneuron subclasses of interneurons, *Pvalb* and *Sst*, after ChABC treatment may result in no net change in GAD67 GABA synthesis.

### ChABC-Induced Juvenile-Like States Are Unlike Classically Induced Homeostatic Plasticity by Bicuculline and TTX

In vivo PNN disruption can induce juvenile ocular dominance plasticity in the mature brain [24] and shift the neural network into an immature state [108]. There are also indications that PNNs are involved in AMPA receptor mobility and may modulate short-term synaptic plasticity in vitro [109]. Importantly, while mature PNNs contribute to an extracellular state which restricts synaptic plasticity, these structures also have a crucial role in synaptic stabilisation [110]. Hence, reduced PNN density throughout development may enable anatomical states in which heightened synaptic flexibility and sprouting is permitted [1], whereas the structural ‘tightening’ of these nets in the mature brain performs a crucial role in both synaptic stabilisation and facilitation of the FS mode of PVB interneurons.

Our aforementioned data suggest that ChABC returns cortical neuronal cultures to a juvenile-like state, in which plasticity may be elevated, but where PVB interneuron FS activity may be impaired. We contrasted these results with well-known modulators of mature synaptic plasticity and network activity, bicuculline and TTX.

Primary cortical cultures are composed mainly of glutamatergic cells, with a smaller proportion of GABA interneurons [111]. Arc is localised to glutamatergic cells, but cFos can be induced in all cell types. While the cell type expressing these markers is not always clear, they do serve as highly informative markers of the state of network activity and resulting plasticity in the cultures. Our data highlight key phenotypic differences in the plasticity induced by bicuculline compared to that of ChABC. Similar to ChABC treatment, bicuculline upregulated expression of *cFos*, an indication of heightened network activity [112]. However, our data also show a distinct upregulation of *Arc* following bicuculline administration which was not present in ChABC-treated cultures. The lack of *Arc* upregulation after ChABC is intriguing given the role of *Arc* in synapse-specific homeostatic plasticity in glutamatergic cells [113], and may indicate that the same synapse-specific homeostatic scaling is not present, or as prominent, in juvenile-like states. Importantly, we found that TTX, a sodium channel blocker, substantially reduced *cFos* compared to vehicle. Expression of *Arc* was also significantly lower in TTX versus bicuculline-treated cultures. In addition, in line with previous findings [54], we found a reduction to *Dlg4* (encoding PSD-95) expression following bicuculline treatment.

Neither bicuculline nor TTX push primary neuronal cultures back to a juvenile state; expression of developmentally regulated genes, *Ascl1*, *Dlx2* and *Kcnc1*, was unchanged by either treatment. However, interestingly, we found a downregulation of *Pax6* following bicuculline treatment. *Pax6* is closely associated with the development of glutamatergic cells, but also plays a role in the mature CNS as a regulator of neurogenesis [114]. Since GABA_A_-R stimulation promotes neurogenesis [115], we interpret the downregulation of *Pax6* by bicuculline as potentially linked to this aspect of GABA_A_-R function.

In addition, we found a substantial upregulation in *Acan* expression following bicuculline, which fits with reports that suggest ACAN is a key activity-dependent component of PNNs [116, 117]. The precise contribution of individual CSPGs to PNN function is not currently known. However, *Acan* appears to be critical in formation of classically recognised mature PNNs, with *Acan* knockout mice exhibiting no WFA labelling [35]. *Acan* knockout in the mouse visual cortex apparently reinstates juvenile, but not mature, ocular dominance plasticity [27]. We suggest that the upregulation of *Acan* after bicuculline may indicate increased ACAN abundance within PNNs, potentially in response to elevated network activity. Consequently, unlike ChABC which appears to induce a more structurally permissive state, this may lead to denser PNNs which promote synaptic stabilisation and confer a more restrictive structural state. However, it should be noted that no reciprocal reduction in *Acan* expression was found in response to TTX-induced network activity reduction.

TTX downregulated expression of *Sema3a* and *Has1*. These components were not significantly affected by bicuculline, which may suggest that the regulation of these does not relate solely to network activity, although further research is required.

Taken together, our data support the idea that ChABC-induced juvenile plasticity enables structural flexibility (as has been observed in the spinal cord [118, 119] and in ChABC targeted axons [37]) and increases network disinhibition primarily via dysregulation of PVB interneuron FS activity. However, we suggest that ChABC-induced plasticity is not akin to bicuculline-regulated mature plasticity, in which we see indications of heightened homeostatic plasticity and, potentially, a bolstering of synaptic stability.

### Juvenile-Like States May Be Related to Alterations in JNK Signalling

Previous evidence has linked the JNK signalling pathway, along with upstream kinases, to key processes in GABAergic interneuron development [72–74, 120] including the regulation of various PNN components throughout development [76]. Moreover, members of the JNK family have been linked to synaptic plasticity [70, 121–125]. Considering this, we investigated the consequences of PNN removal in cortical neuronal cultures on JNK activity, and contrasted this with results from bicuculline and TTX treatment.

We found substantially lower levels of all pJNK isoforms following ChABC treatments. Our previous work has indicated that higher levels of JNK activity, along with upstream kinase TAOK2, are linked to an accelerated developmental phenotype in which PNN density is heightened prematurely [76]. Overall, our data support a role for JNK in ChABC effects. ChABC exposure reduced JNK activity. In previous work [76], we observed that direct inhibition of JNK downregulated *Bcan* and upregulated *Has2* expression—showing very good correspondence to the effects of ChABC. It is interesting that ablation of the PNN itself appears to have a consequence for JNK activity. This suggests a cross-regulation between these two developmentally important players. It is conceivable that if ChABC produces reversion to an immature neuronal state which recapitulates critical period like plasticity, an inherent reduction in JNK activity would be required for this phenotype. PNNs are known to reform in vivo a few weeks after ChABC treatment [25]. Future work may use longer-term timepoint studies of ChABC to better understand the reformation process in vitro. We hypothesise that there may be a coincidence of PNN reformation (e.g. heightened CSPG expression, return of WFA labelling), neuronal maturity and elevated TAOK2/JNK activity. Our experiments measuring *Taok2* expression may provide some support for this hypothesis; *Taok2* expression was heightened following 3 DIV ChABC treatment. Given that *Taok2* is an upstream activator of JNK, this upregulation may be the first sign of a move towards heightened TAOK2-JNK signalling which could contribute to a restoration of PNNs and PVB interneuron maturity.

pJNK levels represent another key difference between ChABC- and bicuculline-induced plasticity states. TTX did not alter levels of pJNK or pERK isoforms. However, bicuculline increased levels of pJNK (particularly, p56 and p54) and pERK (particularly, p42). However, we found no change to the expression of upstream kinase *Taok2* in response to either bicuculline or TTX. We propose this may indicate that the downregulation of pJNK by ChABC owes to the induction of an immature neuronal phenotype, rather than a consequence of or response to increased network activity or plasticity.

The fact that pERK was not downregulated after ChABC fits with the idea that PNN maturation and regulation may interact specifically with the JNK signalling pathway. Indeed, our previous investigation revealed that inhibition of ERK has little effect on the expression of PNN components, with the exception of *Sema3a* [76]. Hence, it is perhaps unsurprising that there is no dysregulation of ERK activity downstream of PNN disruption.

### BDNF Modulates IE Gene Expression in a TAOK-Dependent Manner

BDNF is a crucial player in cortical and hippocampal GABAergic interneuron maturation [59, 107, 126, 127] and has a regulatory role in the closure of the critical period of cortical plasticity and PNN maturation [43–45, 76].

Our data indicated no change in *Bdnf* expression following ChABC treatment. However, as may be expected in a heightened state of synaptic plasticity, *Bdnf* expression was significantly elevated after bicuculline treatment. This result is also in line with other reports which have found that in vivo GABA_A_-R blockade increases cortical and hippocampal BDNF protein levels [128, 129].

BDNF itself is a known modulator of synaptic plasticity. Given that our previous work has revealed many of the accelerating effects of BDNF on interneuron and PNN development are reliant on JNK, we asked whether BDNF modulation of plasticity and interneuron/PNN regulation may also rely on upstream TAOK. To this end, we treated cultures with vehicle, BDNF, TAOK inhibitor (CP-43) or BDNF + CP-43, and measured expression of key PNN, GABAergic and IE genes.

At the treatment time and developmental stage studied, no significant effects of TAOK2 inhibition were detected on *Bcan* or *Has2* expression (Fig. 6) despite the clear downregulatory and upregulatory effects (respectively) of ChABC (Fig. 1), along with reduced JNK activity (Fig. 3). There are around 20 Map3Ks involved in JNK activation. TAOK2 is only one of these, so identical effects of inhibiting TAOK2 and JNK are not expected. In fact, the overlap between the effects of inhibition of TAOK2 and the effects of ChABC is quite limited, whereas the upregulation of TAOK2 by ChABC may imply a role for TAOK2 more in a compensatory response to the induced immaturity.

We found a prominent upregulation of *Ncan*, *Sema3a*, *Gad1*, *Gad2*, *cFos* and *Arc* following BDNF treatment. Interestingly, BDNF effects on *Sema3a*, *cFos* and *Arc* appear to be TAOK reliant, as the upregulation of these genes was negated when CP-43 was administered concomitantly with BDNF. We suggest this indicates that BDNF induces a state of heightened synaptic plasticity and network activity in cortical neuronal cultures, which may be dependent on TAOK signalling. In addition, the observed upregulation of *Gad1* by BDNF treatment mirrors our previous data which show an elevation of GAD67 protein following exogenous BDNF treatment in cortical neuronal cultures [76].

SEMA3A supresses axonal growth while promoting dendritic branching and spine maturation [130, 131]. Moreover, NCAN has been shown to inhibit Sema3F-induced spine elimination [132] and promote neurite outgrowth [133]. The increase in both *Ncan* and *Sema3a* mRNA may relate to a heightened state of dendritic branching and suppression of axonal elaboration which is induced by BDNF and could, at least in part, rely on TAOK signalling.

Previous work has shown that TAOK2 knockdown in cultured neurons reduces neurite branching and increases growth cone collapse [134]. This group have reported similar abnormalities in *Taok2* heterozygous and knockout mice which demonstrate reductions in basal dendrite length, number of basal dendrite spines and dysregulation to spine morphology in these animals [135]. Hence, it is possible that disruption to TAOK2 signalling reduces responsiveness to BDNF-induced dendrite elaboration. This finding may hold clinical significance given the inclusion of *Taok2* in the duplication and deletion of chromosome 16p11.2, copy number variants which have been highly associated with both schizophrenia and autism [136–138].

### Conclusions

Overall, our data have revealed that PNN depletion induces an immature neuronal phenotype which is dissimilar from classically induced mature synaptic plasticity and may be related to JNK signalling. Our results suggest that PNN degradation may be linked with a more permissive environment which is conducive to heightened structural plasticity. The functional consequences of this immature molecular phenotype, and whether this impacts the phenotypic FS activity of PVB interneurons, require further investigation. We suggest that the observed immature molecular phenotype could underpin aspects of the induction of juvenile-like plasticity which has previously been observed following ChABC treatment in vivo*.* Finally, our work further underscores the importance of MAPK signalling downstream of BDNF in the modulatory effects of BDNF on plasticity.

## Supplementary Information

Below is the link to the electronic supplementary material.

Supplementary file1 (PDF 727 kb)

## Data Availability

Original data are available from the corresponding author on reasonable request.
