# Supplementary material for: Enzymatic Degradation of Cortical Perineuronal Nets Reverses GABAergic Interneuron Maturation
Source: Mol Neurobiol. 2022 Mar 1;59(5):2874–93. doi: 10.1007/s12035-022-02772-z (PMC9016038; doi:10.1007/s12035-022-02772-z)
Supplement: Supplementary file 1 — Supplementary file1 (PDF 727 kb) [file 12035_2022_2772_MOESM1_ESM.pdf]

Enzymatic degradation of cortical perineuronal nets reverses GABAergic interneuron maturation

Ashleigh Willis[1], Judith A. Pratt[2], Brian J. Morris[1]

1. Institute of Neuroscience and Psychology, College of Medical Veterinary and Life Sciences, University of Glasgow, G12 8QQ, Glasgow, UK
2. Strathclyde Institute of Pharmacy and Biomedical Sciences, University of Strathclyde, G4 0RE, Glasgow, UK

*Corresponding Author: Professor Brian Morris, brian.morris@glasgow.ac.uk

Supplementary Materials

Target Gene	Forward Primer Sequence (5'->3')	Reverse Primer Sequence (5'->3')
Acan	GTTGCAGACATTGACGAGTGC	AGTCCACCCCTCCTCACATT
Bcan	GATTCCGGGGTCTATCGCTG	ACGACCCCTTTGACCTTGAC
Ncan	AGTATGGGGGCCGGATCTGT	TGGTGTCCTGTGTGTCCTGAT
Vcan	ACCTTCCAACTATCCGGTGC	GGTATGCAGATGGGTTCATGC
Has1	TGTGTCCTGCATCAGTGGTC	TTGGTGAGGTGCCTGTCATC
Has2	GCCATGTGGTTTCACAAGCA	TGAGACCCACTAGCTGGACA
Has3	GCTTCTTTGTGTGGCGTAGC	AGTCCACTGAGTTGCCAAGG
Sema3a	GCCTGTCTTTTCTGGGGTGT	AGCTGTTGGCCAAGCCATTA
Gad1	TTTGGAGCTGTCTGACCACC	AAATCGAGGGTGACCTGTGC
Gad2	TCCTCTCTTGGCTGTAGCTGA	AGAGTTGGCCCTCTCTACTCC
Pvalb	CAAGCAGTCAGCGCCACTTA	GGATGAGCTGGGGTCCATTCT
Sst	CCAACTCGAACCCAGCAATG	TCAGAGGTCTGGCTAGGACA
Dlg4	CTCAAGAGGCGGGTTCAT	AAGCCAAGTCCTTTAGGCCC
cFos	TTTCAACGCCGACTACGAGG	GCGCAAAAGTCCTGTGTGTT
Arc	GAGCGAGAGCTGAAAGGGTT	ACGGTAGAAGACCTCCCTCC
Taok2	AACAGTGCCAGGCAGAAGAG	TCAAGTCCTCTCGAAGCAGGT
Bdnf	CCCGGAGTAGGGATGGAGAA	ATCATGGGCAGTGGAGTGTG
Ascl1	TTCTCCGGTCTCGTCCTACT	CTGCCATCCTGCTTCCAAAGT
Dlx2	TCCTACTCCGCCAAAAGCAG	CTTCCTTGTCCGGCTCGTT
Kcnc1	ACTCAGAGTGACACATGCCC	CCCATTCAGTTTGGAATCTGCT
Pax6	CGGATGAAGCTCAGATGCGA	CTCAAACTCTTTCTCCAGAGCCT

Table S1. List of RT-qPCR primers used in this study.

Figure	ANOVA
1	One way ANOVA<br><br>Main effect of treatment (vehicle or ChABC):<br><br>*Acan*: $F_{(1,11)}$= 0.56, n.s<br>*Ncan*: $F_{(1,11)}$= 0.04, n.s<br>*Vcan*: $F_{(1,11)}$= 0.88, n.s<br>*Sema3a*: $F_{(1,11)}$= 0.00, n.s<br>*Has1*: $F_{(1,11)}$= 0.53, n.s<br>*Has2*: $F_{(1,11)}$= 5.60, p= 0.038<br>*Has3*: $F_{(1,11)}$= 0.10, n.s<br>*Gad1*: $F_{(1,11)}$= 2.47, n.s<br>*Gad2*: $F_{(1,11)}$= 2.20, n.s<br>*Pvalb*: $F_{(1,11)}$= 5.30, p=0.044<br>*Sst*: $F_{(1,11)}$= 7.84, p=0.019<br>*cFos*: $F_{(1,11)}$= 5.52, p= 0.043<br>*Arc*: $F_{(1,11)}$= 0.00, n.s<br>*Dlg4*: $F_{(1,11)}$= 0.72, n.s
2	One way ANOVA<br><br>Main effect of treatment (vehicle, bicuculline or TTX):<br><br>*Acan*: $F_{(2,21)}$= 52.59, p<0.000<br>*Bcan*: $F_{(2,21)}$= 0.06, n.s<br>*Ncan*: $F_{(2,21)}$= 2.44, n.s<br>*Vcan*: $F_{(2,21)}$= 0.62, n.s<br>*Sema3a*: $F_{(2,21)}$= 3.82, p=0.038<br>*Has1*: $F_{(2,21)}$= 4.35, p=0.026<br>*Has2*: $F_{(2,21)}$= 1.12, n.s<br>*Has3*: $F_{(2,21)}$= 1.15, n.s<br>*Gad1*: $F_{(2,21)}$= 2.76, n.s<br>*Gad2*: $F_{(2,21)}$= 4.68, p=0.021<br>*Pvalb*: $F_{(2,21)}$= 0.02, n.s<br>*Sst*: $F_{(2,21)}$= 1.180, n.s<br>*cFos*: $F_{(2,21)}$= 7.58, p= 0.003<br>*Arc*: $F_{(2,21)}$= 15.97, p<0.000<br>*Dlg4*: $F_{(2,21)}$= 4.10, p=0.031
3a-d	One way ANOVA<br><br>Main effect of treatment (vehicle or ChABC):<br><br>pJNK p48: $F_{(1,11)}$= 26.59, p<0.000<br>pJNK p54: $F_{(1,11)}$= 29.06, p<0.000<br>pJNK p56: $F_{(1,11)}$= 37.81, p<0.000<br>pERK p42: $F_{(1,11)}$= 2.54, n.s<br>pERK p44: $F_{(1,11)}$= 1.27, n.s
3e-h	One way ANOVA<br><br>Main effect of treatment (vehicle, bicuculline or TTX):<br><br>pJNK p48: : $F_{(2,21)}$= 2.98, p=0.075<br>pJNK p54: $F_{(2,21)}$= 4.850, p=0.019<br>pJNK p56: $F_{(2,21)}$= 3.685, p=0.043<br>pERK p42: $F_{(2,21)}$= 6.07, p=0.022<br>pERK p44: $F_{(2,21)}$= 4.06, p=0.057

4a-d	One-way ANOVA<br><br>Main effect of treatment (vehicle or ChABC):<br><br>*Ascl1*: $F_{(1,11)}$= 4.85, p=0.037<br>*Dlx2*: $F_{(1,11)}$= 9.02, p=0.012<br>*Kcnc1*: $F_{(1,11)}$= 19.91, p<0.000<br>*Pax6*: $F_{(1,11)}$= 4.71, p=0.020
4e-h	One-way ANOVA<br><br>Main effect of treatment (vehicle, bicuculline or TTX):<br><br>*Ascl1*: $F_{(2,21)}$= 1.24, n.s<br>*Dlx2*: $F_{(2,21)}$= 0.59, n.s<br>*Kcnc1*: $F_{(2,21)}$= 1.93, n.s<br>*Pax6*: $F_{(2,21)}$= 2.43, n.s
5a-b	One-way ANOVA<br><br>Main effect of treatment (vehicle or ChABC):<br><br>*Bdnf*: $F_{(1,11)}$= 2.61, n.s<br>*Taok2*: $F_{(1,11)}$= 4.65, p= 0.054
5c-d	One-way ANOVA<br><br>Main effect of treatment (vehicle, bicuculline or TTX):<br><br>*Bdnf*: $F_{(2,21)}$= 28.25, p<0.000<br>*Taok2*: $F_{(2,21)}$= 1.27, n.s

Table S2. Details of statistical output related to Figures 1 – 5.

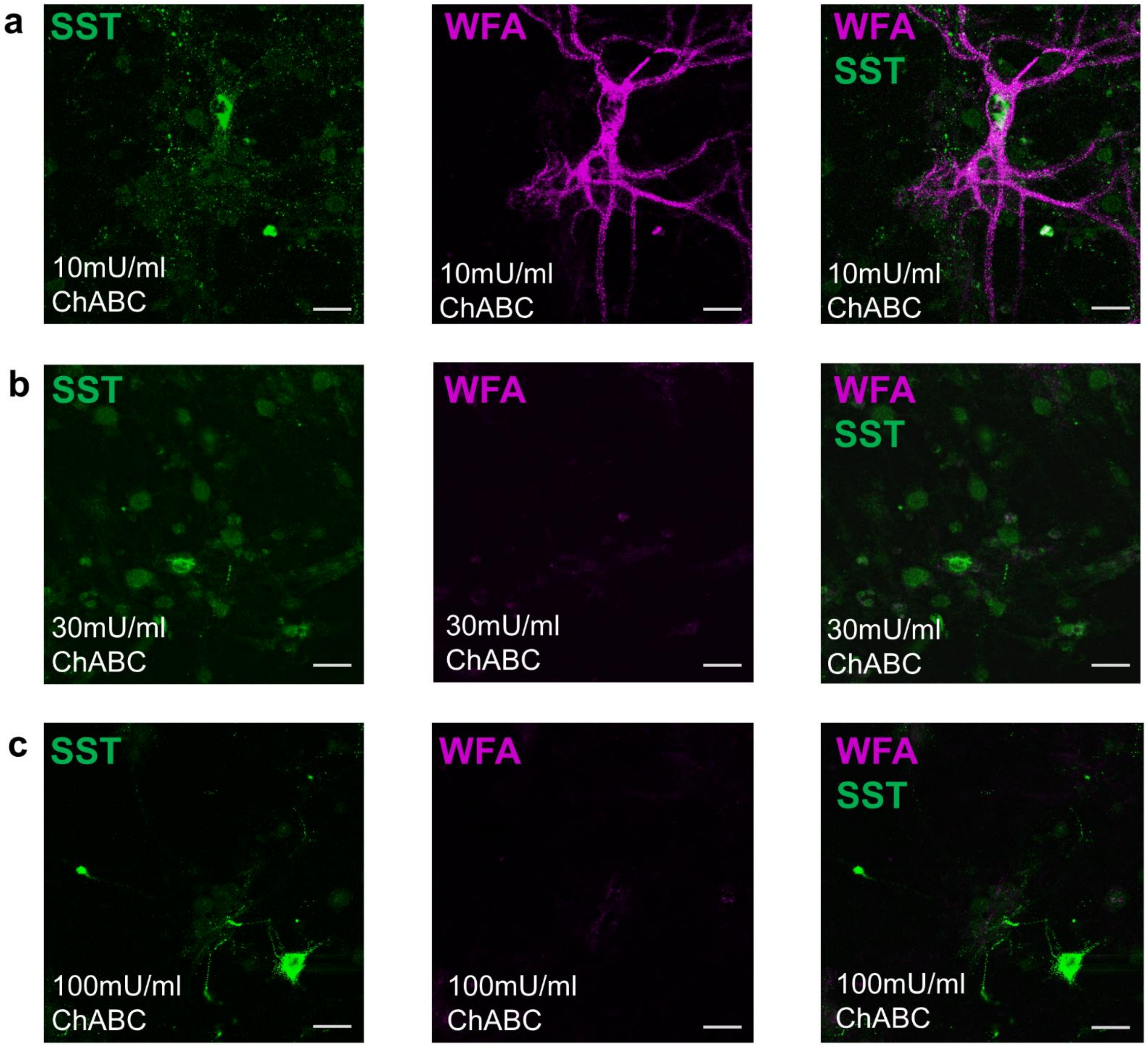

Figure S1. Concentration gradient for ChABC digestion of PNNs. Primary cortical neuronal cultures were treated with either (a) 10mU/ml, (b) 30mU/ml or (c) 100mU/ml ChABC at 18 DIV for 3 DIV. Following treatment, cells were fixed and double-labelled with anti-SST and WFA lectin. WFA labelling was assessed qualitatively via confocal imaging (see methods section **2.3**). PNNs remained present after 10mU/ml ChABC, but were successfully digested with 30mU/ml ChABC. Hence, experiments presented in Figure 1 and Figures 3-5 proceeded to use ChABC at 30mU/ml, applied once at 18 DIV for 3 DIV. N=5 independent cultures/concentration.

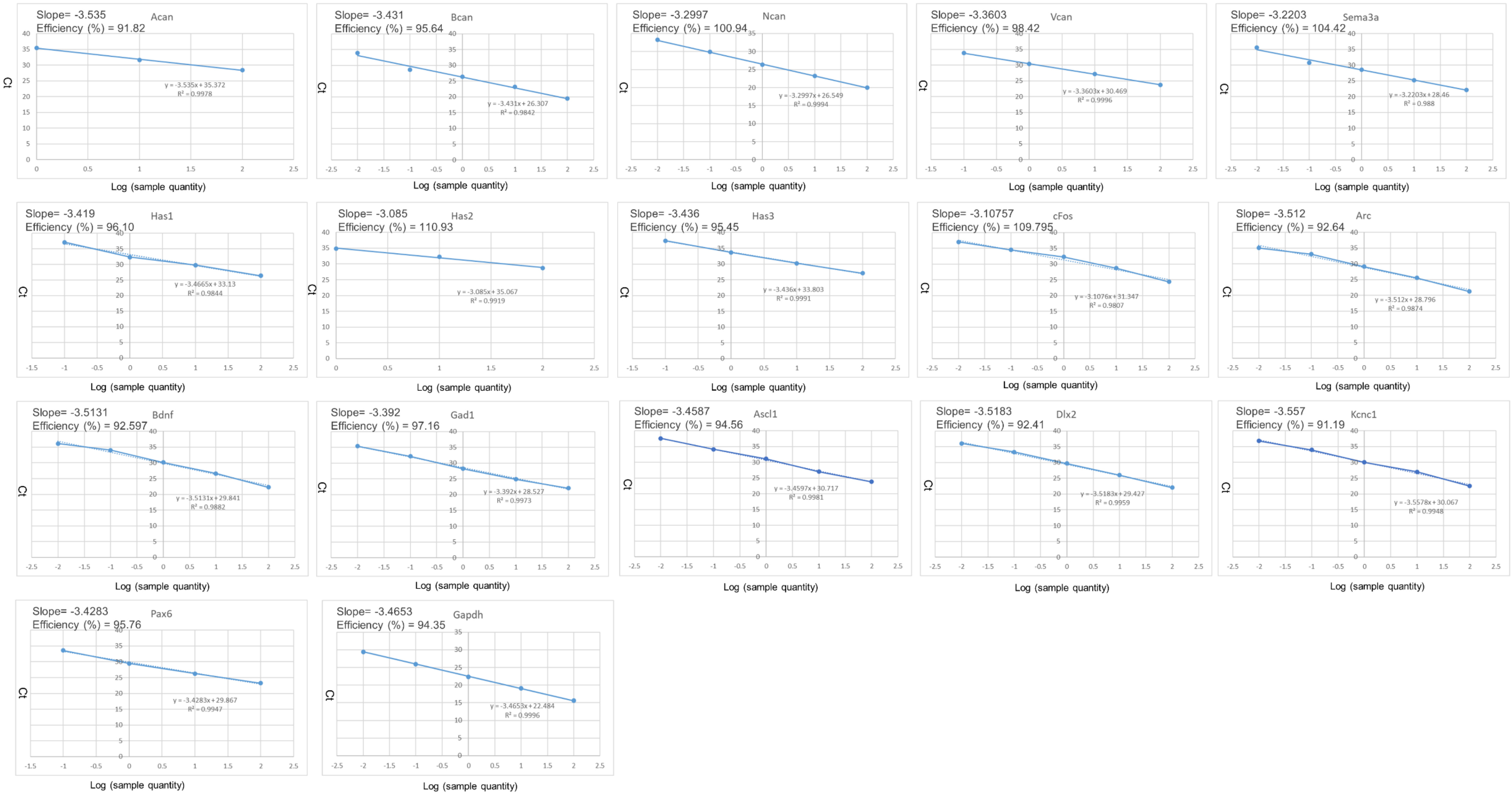

Figure S2. RT-qPCR primer amplifications. Primer amplification curves were produced using cDNA extracted from WT mouse PFC, except for three developmentally regulated genes; cDNA from embryonic cortex (E16) was used for *Ascl1*, *Dlx2* and *Pax6*. Standard curves (1:10 dilution) of cDNA were prepared and subjected to RT-qPCR with each primer (see methods section 2.4). Highest dilution factors for target genes of low expression (*Acan*, *Vcan*, *Has1*,*Has2, Has3* and *Pax6*) were excluded due to signal beyond, or at the limits of (e.g. Ct ~38-39), detection. Primers validated in our previous work are not presented (*Tbp*, *Pvalb, Sst, Gad2* and *Taok2* previously reported as 90-110% efficiency)

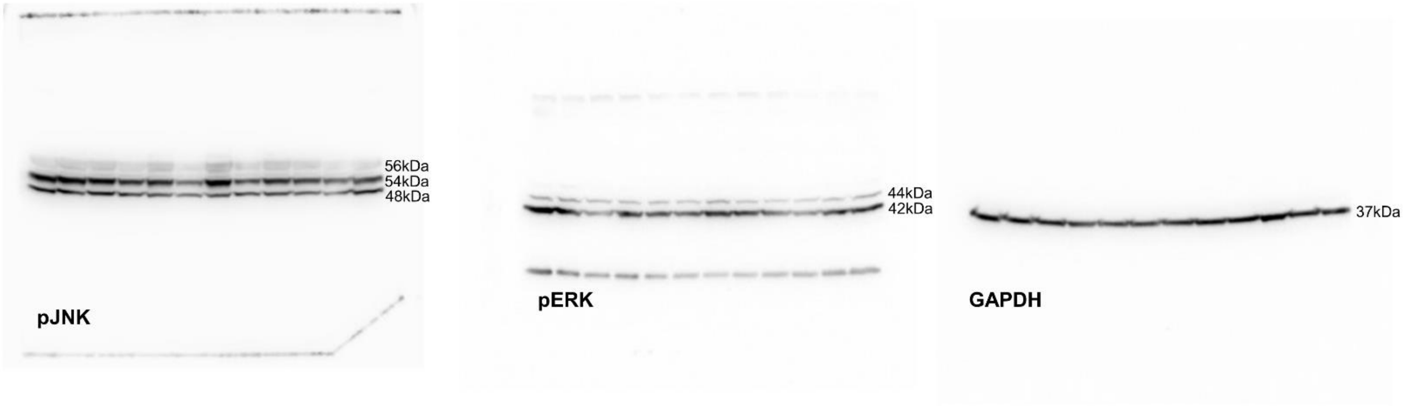

Figure S3. Full length Western blot membrane examples. Full details of protein preparation and immunoblotting protocols can be found the methods section 2.6 and 2.7.
